# Paranoia affected COVID-19 vaccine refusal by increasing beliefs in conspiracy theories and mistrust of science

**DOI:** 10.3389/fpsyg.2025.1718763

**Published:** 2025-12-16

**Authors:** Luca Simione, Monia Vagni, Tiziana Maiorano, Valeria Giostra, Daniela Pajardi, Jan-Willem Van Prooijen

**Affiliations:** 1 Department of International Humanities and Social Sciences, UNINT, Rome, Italy; 2 Institute of Cognitive Sciences and Technologies, CNR, Rome, Italy; 3 Department of Philosophy, Social Sciences, Humanities and Education, University of Perugia, Perugia, Italy; 4 Department of Humanities, University of Urbino, Urbino, Italy; 5 Department of Experimental and Applied Psychology, Vrije Universiteit Amsterdam, Amsterdam, Netherlands

**Keywords:** vaccination hesitancy, paranoia, COVID-19 vaccines, mistrust, psychological factors

## Abstract

**Introduction:**

Psychological determinants of COVID-19 vaccination have been widely studied since the onset of the pandemic, yet little is known about how these factors evolved once vaccines became available. This study addresses this gap by examining psychological predictors of vaccination attitudes and behavior during Italy’s third wave (spring 2021), a phase marked by widespread vaccine access and persistent public uncertainty.

**Methods:**

A total of 375 adults completed standardized measures of anxiety, depression, death anxiety, paranoia, mistrust in science, mistrust in scientific communication, and conspiracy beliefs. Vaccine intentions were assessed through perceived usefulness and safety, and by actual uptake. Using structural equation modeling with Huber-White robust estimation and bias-corrected bootstrapping, we tested a mediation model linking psychological factors to vaccine evaluation and behavior.

**Results:**

The analysis revealed that paranoia exerted a significant indirect effect on both vaccine evaluation and uptake, mediated by mistrust in science and conspiracy beliefs. Death anxiety displayed a small but direct positive association with vaccination behavior.

**Discussion:**

These findings suggest that while contextual factors such as vaccine availability shape overall attitudes, paranoia and related distrust remain stable psychological barriers to vaccine acceptance. The present study extends prior research by clarifying how enduring cognitive biases interact with situational context to influence vaccination decisions, with implications for public health communication strategies.

## 1 Introduction

The mortality rate from COVID-19 has declined since late 2019, largely due to widespread vaccination (Cacciapaglia et al., 2021; Carta et al., 2022). Despite evidence supporting vaccination, hesitancy persists, affecting attitudes toward initial and booster doses (Mahmud et al., 2023; Simione et al., 2022). Initially, factors like death anxiety predicted vaccine propensity when vaccines were unavailable (Bodner et al., 2022; Scrima et al., 2022; Simione et al., 2021). Paranoia, belief in conspiracy theories, and mistrust in science also influenced vaccine intentions (Hornsey et al., 2018; Loomba et al., 2021; Romate et al., 2022; Simione et al., 2021; van Prooijen and Böhm, 2023; see also Knežević et al., 2023; Hughes et al., 2022, for recent large-scale evidence on the structure and predictors of COVID-19 conspiracy beliefs). However, as the pandemic evolved and vaccines became widely available, the social meaning of vaccination and its psychological determinants may have changed. To date, little is known about whether the psychological mechanisms identified early in the pandemic remain stable or shift once vaccines are accessible, and few studies have explored these dynamics within later pandemic phases, when both psychological distress and perceived risk of infection declined (Manchia et al., 2022). Moreover, most previous studies have focused on vaccination intentions rather than actual vaccination behavior, leaving open questions about how attitudes translate into real-world actions. Addressing these gaps, we hypothesized potential changes in the impact of psychological factors on vaccine attitudes as the distress response evolved during the pandemic. Building on previous models of vaccine hesitancy (Anakpo and Mishi, 2022; Bronstein et al., 2022; De Coninck et al., 2021; Scrima et al., 2022; Simione et al., 2021), we aimed to replicate findings from Simione et al. (2021), which included death anxiety, trait anxiety, depression, and paranoia as predictors of vaccination intentions, with conspiracy beliefs and mistrust in medicine and science as mediators. With vaccines already disseminated, our study measured both vaccine evaluation and actual uptake, providing insights into attitudes and psychological causes of vaccine hesitancy.

Vaccine hesitancy reflects attitudes toward vaccines that can be characterized by uncertainty about getting vaccinated. Vaccine hesitancy differs from refusal, indicating instead a position against getting vaccinated. In this framework, vaccine propensity is predicted by both explicit and implicit attitudes toward vaccines (Simione et al., 2022), which in turn are linked to actual vaccine behavior (Hornsey et al., 2018). Therefore, assessing both attitudinal and behavioral dimensions provides a more comprehensive understanding of vaccine hesitancy, as previous studies on COVID-19 or other vaccines mostly rely only on the intention to get vaccinated (Caserotti et al., 2021; Scrima et al., 2022; Simione et al., 2021; see also Hornsey et al., 2018), while only few studies analyzed vaccination intentions in concert with vaccination behavior (e.g., Graupensperger et al., 2021). Regarding other diseases, several previous studies analyzed vaccine uptake and vaccination attitudes (Byrne et al., 2012; Gowda et al., 2012; Greyson et al., 2021; Rosenthal et al., 2008). Overall, these studies confirmed the pattern reported in intention-based studies, highlighting the effects of trustworthiness and information on vaccination decisions (Byrne et al., 2012), as well as the role of general vaccine attitudes in predicting specific vaccine behaviors (Greyson et al., 2021; Rosenthal et al., 2008). Moreover, they generally found a positive correlation between the intention of getting vaccinated, vaccine evaluation, and actual behavior (Byrne et al., 2012; Greyson et al., 2021; Rosenthal et al., 2008).

In the first pandemic wave, when vaccines were not yet available, psychological variables linked to death anxiety or existential concerns were identified as predictor of vaccine propensity (Bodner et al., 2022; Scrima et al., 2022; Simione et al., 2021). Existential anxiety, referring to the awareness of one’s mortality and the search for meaning under threat (Pyszczynski et al., 2021), was found to positively drive vaccination intention, as vaccines would decrease the likelihood of dying from COVID-19 (Pyszczynski et al., 2021; Waite et al., 2022). People tended to propend for vaccination in the phases when the perceived risk of infection was highest, while hesitancy increased when infections appeared less dangerous (Caserotti et al., 2021). Moreover, increased social restrictions usually accompanied the infection waves, heightening existential concerns. These concerns may also foster susceptibility to conspiracy thinking, linking mortality awareness to mistrust toward science and institution (van Prooijen and Lange, 2014; van Prooijen, 2019), with the paradoxical effect of reducing the intention to get vaccinated (Scrima et al., 2022; Simione et al., 2021).

Paranoia, conspiracy beliefs, and mistrust in science represent interrelated but distinct constructs underlying vaccine hesitancy. Since the first spread of COVID-19, conspiracy theories mainly focusing on the virus’s alleged artificial origin and the harmfulness of vaccines have circulated widely (Sallam et al., 2021). However, recent analyses suggest that the perceived popularity of such conspiracy theories may have been overestimated, underscoring the need for distinguishing general skepticism from clinically significant distrust (Sutton and Douglas, 2022). Understanding the antecedents and consequences of such beliefs is crucial, given their association with vaccine hesitancy or refusal (van Mulukom et al., 2022). Among other psychological factors, one of the most consistent predictors of believing in conspiracy theories and anti-vaccine attitudes is paranoia (Simione et al., 2021). Paranoia, as defined by van Prooijen and Lange (2014), involves self-relevant suspicious beliefs about others’ malicious intentions (Imhoff and Lamberty, 2018). At subclinical levels, paranoia fosters the perception of threat and intentional harm, which may generalize to distrust in scientific authorities and health institutions. Although paranoia and conspiracy beliefs are strongly correlated, they are distinct constructs: paranoia targets potential personal harm, while conspiracy beliefs attribute negative intentions to powerful groups affecting society (Imhoff and Lamberty, 2018). Conspiracy beliefs are significant predictors of vaccine hesitancy, particularly among individuals with high fear and low trust (Anakpo and Mishi, 2022; De Coninck et al., 2021).

Mistrust in science, defined as generalized skepticism toward scientific expertise and its communication, has also emerged as a major determinant of COVID-19 vaccine attitudes (Romate et al., 2022). This mistrust is linked to concerns about the information provided by authorities, skepticism toward vaccine-developing companies, pharmaceutical lobbying, and policymakers’ motives (Murphy et al., 2021). It is often reinforced by doubts about the healthcare system’s ability to manage the pandemic (Simione and Gnagnarella, 2020; Simione et al., 2021). Distrust in science is also associated with conspiracy beliefs (Bertin et al., 2020; Hornsey et al., 2023; Zhang et al., 2021) and paranoia (Simione et al., 2021; van Prooijen and Douglas, 2018), contributing to negative attitudes toward vaccines (Antinyan et al., 2021; Murphy et al., 2021; Nazlı et al., 2022; Simione et al., 2021) and was considered a key factor in vaccination refusal during the first pandemic wave (Bajos et al., 2022; Seddig et al., 2022). Overall, paranoia, conspiracy beliefs, and mistrust in science form a cluster of distrust-related factors consistently associated with vaccine hesitancy.

While much of the existing evidence comes from international research, studies conducted in Italy offer a valuable opportunity to examine these psychological dynamics within a socio-cultural context marked by high early mortality rates, strong institutional communication, and intense public debate over vaccination. In a study on vaccine attitudes during Italy’s first pandemic wave, Simione et al. (2021) found that mistrust of science and conspiracy beliefs negatively correlated with vaccine intention. Anxiety, death anxiety, depression, and paranoia emerged as key psychological predictors. Trait anxiety increased vaccine propensity by reducing conspiracy beliefs, while death anxiety had the opposite effect. Depression and paranoia decreased vaccine propensity by increasing mistrust in medical science. This complex interplay of psychological factors influenced vaccine attitudes both directly and indirectly. Given changes in societal and psychological conditions during later pandemic waves (Manchia et al., 2022; Vagni et al., 2022), these patterns may not hold over time. Additionally, the study initially assessed only vaccine propensity, not actual behavior, as vaccines were unavailable to the general population then.

Building on these findings, the present study investigates whether the psychological mechanisms identified during the first wave persisted or changed when vaccines became widely available. We conducted a new cross-sectional study in spring 2021, during the third wave of COVID-19 infections in Italy, to test the model from Simione et al. (2021). By then, vaccination was available but not mandatory in Italy, but the government was actively promoting it. With vaccines being widely available, we measured two outcomes: COVID-19 vaccine attitudes and actual vaccine behavior (i.e., whether participants received the vaccine). Based on the literature, we hypothesized a strong relationship between vaccine behavior and vaccine attitudes. We also expected mistrust of science and conspiracy beliefs, as assessed by the Beliefs on COVID-19 (BOC-19) scale, to correlate with negative vaccine attitudes and lower likelihood of vaccination, as supported by recent work linking dispositional mistrust and conspiracy beliefs to vaccine-related outcomes (Knežević et al., 2023; Hughes et al., 2022). We further hypothesized that death anxiety, trait anxiety, and depression would be less predictive of vaccine attitudes and behavior due to their reported decline over successive pandemic waves, whereas paranoia would remain a stable predictor of negative vaccine evaluation and behavior. By testing these relationships across different pandemic phases, this study contributes novel evidence on the persistence of distrust-related psychological processes in vaccine hesitancy.

## 2 Materials and methods

### 2.1 Participants

The study sample included 375 Italian participants (303 females and 72 males, mean age = 39.38 years, SD = 13.56), enrolled via social media using convenience sampling rather than random sampling. Level of education, assessed as number of years spent in education, was on average 18.01 years (SD = 2.78). Thirteen participants (3.5%) reported psychological or psychiatric conditions such as general anxiety or depression. Regarding religious beliefs, 175 participants (47%) declared to be agnostic or atheist, 61 participants (16%) declared to be non-practicing Catholics, and 139 participants (37%) declared to be practicing Catholics. Regarding the working condition of participants, 66 (18%) were students, 171 (45%) were self-employed workers, 118 (31%) were employees, 5 (2%) were unemployed, and 15 (4%) were pensioners. Regarding COVID-19 vaccination, 290 participants (77%) were vaccinated while 85 (23%) were not. The vast majority of vaccinated participants received the Comirnaty (Pfizer-BioNTech) or Vaxzevria (Astrazeneca) vaccines, respectively 70 and 27%, and only a few received the Moderna or Janssen (Johnson & Johnson) vaccines, about 3%.

This study was part of a larger project in which participants compiled a battery of questionnaires and then performed an implicit attitudes task. For the purposes of the present contribution, only the results of the questionnaires were analyzed and reported.

### 2.2 Procedure

The questionnaires were administered online with PsyToolkit (Stoet, 2017). The items were presented in a series of successive modules after participants read and signed the informed consent to participate in the study. The order of questionnaire modules was consistent for all participants: demographics first, followed by psychological measures, and finally vaccine-related questions. At the end, participants reported vaccination status and experience. All data was collected anonymously. Informed consent was obtained from all participants. Ethical approval for this study was granted by the Ethics Committee of the University of Urbino and all procedures performed were under the ethical standards of the 1964 Helsinki Declaration.

### 2.3 Materials

Sociodemographic information collected included sex, age, education level in years, nationality, relationship status, number of children, religious beliefs, working conditions, income changes during the last year, and presence of psychological conditions.

The psychological measures included in this study (DASS-21, ECQ death anxiety subscale, SCL-90-R paranoia subscale, and BOC-19) were selected to maintain consistency with Simione et al. (2021), allowing direct replication and extension of the previously established model of psychological predictors of vaccine attitudes. Psychological symptoms were assessed using the 21-item version of the Depression, Anxiety, and Stress Scale, DASS-21 (Lovibond and Lovibond, 1995). Each item is evaluated on a 4-point Likert scale. DASS-21 includes three subscales of 7 items each measuring depression (e.g., “I felt down-hearted and blue”), anxiety (e.g., “I felt scared without any good reason”), and stress (e.g., “I found myself getting agitated”). In our sample, the three subscales showed good reliability, with Cronbach’s α, respectively, of 0.90, 0.83, and 0.90.

Death anxiety was assessed with the 5-item death anxiety subscale of the Existential Concerns Questionnaires, ECQ (van Bruggen et al., 2017). Items measured participants’ fear of death (e.g., “It frightens me that at some point in time I will be dead”), illness (e.g., “I worry that, out of the blue, something terrible might happen to me”), and unpredictability of life (e.g., “I become anxious when I realize how vulnerable my body is to the dangers of life”), and were evaluated on a 5-point Likert scale. This scale showed excellent internal reliability, Cronbach’s α = 0.90.

Paranoia was assessed with the 6-item subscale for paranoid ideation of the Symptoms Checklist 90 Revised, SCL-90R (Prunas et al., 2012), which considered paranoid behavior as fundamentally a disordered mode of thinking characterized by projective thought and suspiciousness. Then, this scale evaluated the presence of a distrustful attitude toward others (e.g., “Feeling that most people cannot be trusted”) and ideas of reference (e.g., “Feeling that you are watched or talked about by others”). Each item reported one symptom whose presence was evaluated on a 5-point Likert scale. The scale showed excellent internal reliability in our sample, Cronbach’s α = 0.88.

Then, the Belief on COVID-19 scale was administered, BOC-19 (Simione et al., 2021). This scale includes 11 items, evaluated on a 5-point Likert scale, which are divided into three subscales, i.e., belief in conspiracy theories (BCT), mistrust in medical information (MMI), and mistrust of medicine and science (MMS). The three subscales reported good internal reliability in our sample, with Cronbach’s α, respectively, of 0.80, 0.75, and 0.78.

After completing the questionnaires, participants reported if they were vaccinated against COVID-19, the type of vaccine, and their degree of agreement with the following statement: “I think the vaccine I took is safe and I would do it again.” Vaccine evaluation was reported on a 5-point Likert scale ranging from 1, “completely disagree,” to 5, “completely agree.” Instead, participants who did not take the COVID-19 vaccine reported which vaccine they would prefer and their degree of agreement with the following statement: “I think vaccination is safe and I would like to do it as soon as possible.” Participants also reported their experiences with COVID-19, including: if they ever had COVID-19, if they lost someone to COVID-19, if they were hospitalized due to COVID-19, etc. Self-report measures were used for feasibility, though potential social desirability and recall bias should be noted.

### 2.4 Data analysis

First, demographic variables were coded as numbers for the successive analyses. Sex was coded as 0 = female, 1 = male. The psychological condition was coded as 0 = absent, 1 = present. Religious beliefs were coded as 0 = atheist or agnostic, 1 = non-practicing Catholic, and 2 = Catholic. Age and education level were already on a continuous scale as they were expressed in years.

The presence of common method bias (Carta et al., 2022) was checked through Harman’s one-factor test and the correlation matrix procedure. In the first test, a single-factor exploratory model including all the items administered was conducted: a proportion of the variance explained by this single factor higher than 50% would be suggestive of the presence of bias. The second test would be interpreted as indicative of common method bias if a correlation higher than 0.90 was present between any pair of variables. Then, the three-factor structure of the BOC was checked with a confirmatory factor analysis (CFA). The model was evaluated for its goodness of fit indexes including relative chi-square (χ2/df), comparative fit index (CFI), root mean square error of approximation (RMSEA) with related 90% confidence intervals, and standardized root mean square residual (SRMR). Model fit was considered adequate with the following values: relative χ^2^ < 5.00, CFI > 0.90, RMSEA < 0.06, SRMR < 0.08 (Hu and Bentler, 1999).

**Figure 1:**
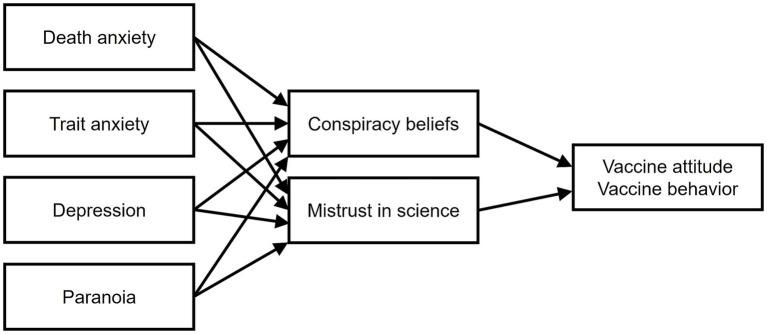
The theoretical model tested. The covariates (sex, age, education level, religious beliefs) and the direct path from the psychological variables (on the left) to the outcome are not shown for sake of clarity.

Bivariate correlations and unpaired *t*-tests compared vaccinated and unvaccinated participant, reporting effect size as Cohen’s d. The primary model (Figure 1) was designed based on the first pandemic wave (Simione et al., 2021) and included death anxiety, anxiety, depression, and paranoia as predictors, BCT and MMS as mediators, and vaccine attitude as outcome. Covariates included sex, age, education, and religious belief. This mediational model is fully saturated, meaning that all possible paths among the included variables were estimated, so the model reproduces the observed data perfectly; therefore, no global fit indices could be evaluated, and the focus is on the significance and size of individual paths and mediation effects. The mediation model was tested through structural equation modeling conducted by means of maximum likelihood estimation. Huber-White robust standard error estimator was applied to exclude bias due to heteroscedasticity. Mediated or indirect paths were tested with bias-corrected bootstrapped confidence intervals. Unstandardized coefficients (b) and their confidence intervals (CI) were reported for each significant path, along with the standardized coefficient (β). The model included 33 estimated parameters, and this made our results interpretable with at least 330 participants per group, while the sample involved in this analysis had 375 participants. The same model was then conducted with the vaccine behavior as the outcome. As vaccine behavior was a binary variable, a diagonally weighted least squares (WLSMV) estimator was implied, a robust estimator which does not assume normally distributed variables, and it is specifically designed for ordinal data.

We conducted two additional control analyses to examine the robustness of our primary models. First, we tested models on vaccine attitude and vaccine behavior in which the DASS stress scale was also added as a predictor. Second, we added the BOC-19 factor of MMI to the two models on vaccine attitude and vaccine behavior, which was not included in the main analysis. These control analyses allowed us to verify the stability of our findings while incorporating additional variables which had been assessed in our dataset but were not considered in the original Simione et al. (2021) model. This allowed us to evaluate their potential role as additional factors and to determine whether their inclusion altered the other paths in the models. All our analyses were conducted with R statistical software (R Core Team, 2014) and mediation models by means of the lavaan package (Rosseel, 2012).

## 3 Results

### 3.1 Preliminary analyses

The first analysis checked the presence of a common method bias. Both the Harman’s test and the correlation matrix test indicated the absence of a common method bias. Specifically, the single-factor model explained only 18% of the variance, well below the 50% threshold, and correlations among variables ranged from 0.01 to 0.63, suggesting no substantial method bias.

The second analysis checked the factor structure of the BOC-19 scale. A CFA analysis on BOC-19 items was conducted using maximum likelihood estimation with robust (Huber-White) standard errors. The three-factor model proposed in Simione et al. (2021) showed inadequate fit indices, relative χ^2^ = 7.71, CFI = 0.83, RMSEA = 0.13 (90% confidence interval 0.12 to 0.15), and SRMR = 0.10. Modification indices indicated that correlating conceptually related item errors (items 1–2, 5–9, 8–9, 10–11) improved model fit, reflecting the overlapping content of items rather than measurement flaws. After including these correlated errors, the model fit improved substantially, relative χ^2^ = 2.81, CFI = 0.96, RMSEA = 0.07 (90% confidence interval: 0.05 to 0.08), and SRMR = 0.06. Although item 11 showed a low loading, b = 0.25, it remained significant and did not compromise the overall scale validity. All other paths from items to their respective factors were significant at *p* < 0.001 and of large magnitude, b > 0.66. The model with correlated error terms outperformed the first one without correlated errors, Δχ^2^ = 469.22. Overall, this CFA supports the three-factor BOC-19 structure with acceptable fit after accounting for correlated items.

### 3.2 Correlation analysis

Bivariate Pearson correlations were computed to examine relationships among demographic, psychological, and BOC-19 variables. We also considered practical relevance alongside statistical significance. As shown in Table 1, age was negatively correlated to all psychological symptoms but positively correlated with beliefs in COVID-19 conspiracy theories and mistrust in medicine and science. Female sex predicted higher death anxiety. Education was negatively correlated with all psychological symptoms and with the BCT scale. Religious beliefs were related to decreased paranoia and increased conspiracy beliefs. Lastly, the presence of a psychological condition was correlated with anxiety, depression, and paranoia.

**Table 1:** Correlations between demographics and other variables.

Variable	Sex	Age	Education	Religious beliefs	Psychological condition
DASS anxiety	0.05	−0.28**	−0.35**	−0.01	0.28**
DASS depression	0.01	−0.25**	−0.32**	−0.07	0.24**
ECQ death anxiety	0.18**	−0.19**	−0.17**	−0.03	0.07
SCL-90 paranoia	−0.03	−0.25**	−0.43**	−0.12*	0.24**
BOC-19 BCT	−0.03	0.20**	−0.13**	0.11*	−0.03
BOC-19 MMI	0.05	0.02	0.05	0.07	−0.02
BOC-19 MMS	0.03	0.20**	0.08	0.04	0.03
Vaccine evaluation	−0.05	−0.10*	0.01	−0.03	0.01

* indicates *p* < 0.05. ** indicates *p* < 0.01.

Afterward, we assessed correlations between psychological variables and BOC-19 scores. Table 2 reported the correlation coefficients with their levels of significance. As shown, all psychological variables were strongly and positively correlated. All the BOC-19 scores were positively and strongly correlated with each other as well. However, none of them were related to the BOC-19 scores except for the paranoia, which showed weak but significant positive correlations with both BCT and MMS. BOC-19 scores were related to more negative vaccine attitudes. These patterns suggest that paranoia was a central psychological predictor, whereas psychological or existential distress had limited impact on COVID-related beliefs.

**Table 2:** Correlations between psychological variables, BOC-19 scores, and vaccination.

Variable	1	2	3	4	5	6	7
1. DASS anxiety	–						
2. DASS depression	0.63**	–					
3. ECQ death anxiety	0.37**	0.41**	–				
4. SCL-90 paranoia	0.46**	0.59**	0.43**	–			
5. BOC-19 BCT	0.02	0.04	0.02	0.14**	–		
6. BOC-19 MMI	−0.01	0.07	0.10	0.09	0.40**	–	
7. BOC-19 MMS	0.01	0.06	0.02	0.11*	0.54**	0.54**	–
8. Vaccine evaluation	−0.02	−0.01	−0.01	−0.08	−0.51**	−0.42**	−0.52**

* indicates *p* < 0.05. ** indicates *p* < 0.01.

### 3.3 Comparison between vaccinated and non-vaccinated participants

In this section, we report the comparisons between non-vaccinated (coded as 0) and vaccinated (coded as 1) participants. Regarding demographic variables, vaccinated participants were older (M0 = 34.74, M1 = 40.74, *t*(130.41) = 3.52, *p* < 0.01, *d* = 0.44) and had a higher education level (M0 = 16.19, M1 = 18.54, *t*(117.08) = 6.51, *p* < 0.01, *d* = 0.85). No differences were found on presence of psychological conditions (M0 = 0.04, M1 = 0.03, *t*(135.36) = 0.03, *p* = 0.097, *d* = 0.01), nor for religious beliefs (M0 = 0.93, M1 = 0.90, *t*(135.38) = 0.29, *p* = 0.77, *d* = 0.04).

Regarding psychological variables, vaccinated participants reported small but significant lower level of depression (M0 = 12.86, M1 = 10.66, *t*(112.62) = 3.68, *p* < 0.01, *d* = 0.48) and anxiety (M0 = 10.59, M1 = 9.06, *t*(114.98) = 3.45, *p* < 0.01, *d* = 0.45) as compared to non-vaccinated ones, whereas they did not differ in death anxiety (M0 = 6.43, M1 = 5.94, *t*(118.68) = 0.84, *p* = 0.40, *d* = 0.11). They instead showed a large effect on paranoia, with non-vaccinated participants reporting more than twice the score of vaccinated ones (M0 = 12.07, M1 = 5.95, *t*(103.24) = 5.78, *p* < 0.01, *d* = 0.80).

Comparisons also revealed a moderate effect on beliefs in conspiracy theories (M0 = 10.08, M1 = 7.35, *t*(115.49) = 5.25, *p* < 0.01, *d* = 0.70) and a medium effect on mistrust in science (M0 = 15.27, M1 = 13.43, *t*(119.63) = 3.63, *p* < 0.01, *d* = 0.47), with non-vaccinated participants reporting higher scores than vaccinated ones. They instead did not significantly differ in their mistrust of medical information (M0 = 7.32, M1 = 6.90, *t*(134.35) = 1.51, *p* = 0.13, *d* = 0.19). Lastly, the two groups reported a moderate difference in the vaccine attitude, with vaccinated participants reporting a more positive evaluation than non-vaccinated ones (M0 = 3.25, M1 = 4.17, *t*(108.19) = 4.92, *p* < 0.01, *d* = 0.66). These results underscore the importance of paranoia and conspiracy beliefs in shaping vaccination outcomes, beyond demographic differences.

### 3.4 Mediation model

We applied the Simione et al. (2021) mediation model to the current sample to assess whether previous patterns held during the third pandemic wave. This approach allows replication while testing robustness under different contextual conditions. The model included the psychological variables as antecedent variables, the BOC-19 factors of BCT and MMS as mediators, and the vaccine attitudes as outcome (see Figure 1). Unstandardized coefficients (β) with their standard error and bootstrapped confidence intervals were reported in Table 3, along with the standardized coefficients (b). In the model, the only psychological variable which was significantly related to both BCT and MMS was paranoia. In turn, both BCT and MMS significantly predicted reduced vaccine evaluation. All the other paths from the psychological variables to BOC-19 scores and the outcome were not significant. The test of indirect effects revealed that paranoia reduced vaccine evaluation through its positive effect on BCT (*b* = −0.02, CI = [−0.02, −0.01], β = −0.07) and on MMS (*b* = −0.02, CI = [−0.02, −0.01], β = −0.06). The effect of paranoia on vaccine evaluations was fully mediated by the BOC-19 factors.

**Table 3:** SEM estimated coefficients for model on vaccine evaluation.

Path	*b*	SE	CI_lower_	CI_upper_	β	*p*
Depression → BCT	−0.02	0.06	−0.14	0.11	−0.02	0.74
Anxiety → BCT	−0.02	0.08	−0.18	0.15	−0.02	0.82
Death anxiety → BCT	−0.03	0.06	−0.16	0.08	−0.03	0.64
Paranoia → BCT	0.11	0.04	0.03	0.20	0.20	<0.01
Depression → MMS	0.04	0.07	−0.09	0.19	0.05	0.54
Anxiety → MMS	−0.01	0.09	−0.17	0.18	−0.01	0.88
Death anxiety → MMS	−0.03	0.06	−0.14	0.08	−0.03	0.66
Paranoia → MMS	0.10	0.04	0.03	0.18	0.19	<0.01
BCT → VaccEval	−0.11	0.02	−0.15	−0.07	−0.34	<0.01
MMS → VaccEval	−0.12	0.02	−0.16	−0.08	−0.34	<0.01
Depression → VaccEval	0.02	0.02	−0.02	0.05	0.05	0.39
Anxiety → VaccEval	−0.01	0.02	−0.06	0.03	−0.04	0.52
Death anxiety → VaccEval	0.01	0.02	−0.02	0.03	0.02	0.71
Paranoia → VaccEval	0.01	0.01	−0.02	0.02	−0.01	0.88

VaccEval = Vaccine evaluation. b = unstandardized coefficient, SE = standard error, CI_lower_ and CI_upper_ = bootstrapped confidence intervals, β = standardized coefficient, p = significant level. Significant paths are reported in boldface.

For vaccine behavior, the same model revealed a partial mediation: paranoia directly predicted lower vaccine uptake, in addition to indirect effects through (*b* = −0.01, CI = [−0.01, −0.01], β = −0.04) and MMS (*b* = −0.01, CI = [−0.01, −0.01], β = −0.03). Coefficients for all paths of this model were reported in Table 4. Death anxiety also showed a small positive direct effect on behavior, suggesting situational relevance for actual vaccination.

**Table 4:** SEM estimated coefficients for model on vaccine behavior.

Path	*b*	SE	CI_lower_	CI_upper_	β	*p*
Depression → BCT	−0.02	0.06	−0.13	0.12	−0.02	0.73
Anxiety → BCT	−0.02	0.08	−0.18	0.17	−0.02	0.83
Death anxiety → BCT	−0.03	0.06	−0.14	0.08	−0.03	0.63
Paranoia → BCT	0.11	0.04	0.03	0.19	0.20	<0.01
Depression → MMS	0.04	0.07	−0.09	0.19	0.05	0.52
Anxiety → MMS	−0.01	0.09	−0.16	0.18	−0.01	0.87
Death anxiety → MMS	−0.03	0.06	−0.14	0.09	−0.03	0.65
Paranoia → MMS	0.10	0.04	0.02	0.18	0.19	<0.01
BCT → VacBehav	−0.02	0.01	−0.03	−0.01	−0.19	<0.01
MMS → VacBehav	−0.01	0.01	−0.03	0.01	−0.13	<0.05
Depression → VacBehav	0.01	0.01	−0.02	0.01	0.01	0.97
Anxiety → VacBehav	−0.01	0.01	−0.02	0.01	−0.04	0.48
Death anxiety → VacBehav	0.01	0.01	0.00	0.02	0.13	<0.01
Paranoia → VacBehav	−0.02	0.01	−0.02	−0.01	−0.26	<0.01

VacBehav = Vaccine behavior. b = unstandardized coefficient, SE = standard error, CI_lower_ and CI_upper_ = bootstrapped confidence intervals, β = standardized coefficient, p = significant level. Significant paths are reported in boldface.

Control analyses incorporated scales not included in the original model: DASS stress and BOC-19 MMI. These analyses were conducted to test robustness, as these variables were assessed but excluded from Simione et al. (2021). Including either stress or MMI did not meaningfully change the primary mediation patterns, reinforcing the central role of paranoia. For full coefficients (see Supplementary Table S1). Overall, results highlight paranoia as the most practically relevant predictor of vaccine attitudes and behavior, with full mediation for attitudes and partial mediation for behavior, emphasizing the theoretical and applied significance of these psychological factors.

## 4 Discussion

The principal aim of this study was to examine the relationship of several psychological variables with attitudes toward COVID-19 vaccination and the propensity to get vaccinated during the third wave of COVID-19 infections, i.e., when the vaccines were available for most of the Italian population. We based our research on previously presented models of COVID-19 vaccine hesitancy (Anakpo and Mishi, 2022; Bronstein et al., 2022; De Coninck et al., 2021; Scrima et al., 2022; Simione et al., 2021). In particular, we aimed to replicate the findings reported in Simione et al.’s model (2021), tested during the first wave of COVID-19 infection and which included death anxiety, trait anxiety, depression, and paranoia as predictors of vaccination intentions, and belief in conspiracy theories (BOC-19 BCT) and mistrust of medicine and science (BOC-19 MMS) as mediators. As the psychological status of individuals changed during the subsequent different pandemic waves, with an overall decrease in psychological distress after the first wave (Manchia et al., 2022), we hypothesized that the effect of such variables on vaccine attitude could have changed. Partially consistent with our expectation, we obtained no significant relationship with vaccine attitudes for death anxiety, trait anxiety, and depression, while only death anxiety had a significant positive relationship with vaccine behavior. However, as hypothesized, we found that paranoid ideation was related to increased mistrust in science and conspiracy beliefs, and to decreased vaccine attitude and vaccine behavior as mediated by them. Correlational analyses confirmed that only paranoia, among the psychological variables examined, was linked to increased conspiracy beliefs and mistrust. This pattern suggests that stable personality traits, rather than transient emotional states, may play a central role in shaping vaccine hesitancy.

The main finding of this study was the central role of paranoia in the mediation model, as it predicted vaccine attitudes and behavior through BOC-19 factors: paranoia was related to a poor evaluation of COVID-19 vaccines and reduced vaccine behavior through increased beliefs in conspiracy theories and mistrust of science. Larsen et al. (2021) found that COVID-19 conspiratorial thinking was associated with higher levels of uncertainty and paranoia, highlighting the importance of paranoia in the development of conspiratorial thinking during a pandemic. These results align with cognitive models of paranoia, suggesting that threat perception and attribution biases contribute to distrust of authorities and medical institutions, and that vaccine hesitancy and refusal are significantly associated with paranoia and conspiracy beliefs (Nazlı et al., 2022). Prior literature supports these associations (Jolley and Paterson, 2020; Kuhn et al., 2021; van Mulukom et al., 2022), and our findings extend them by showing a direct effect of paranoia on actual vaccination behavior, not only intentions, as showed by the large effects obtained in the analysis comparing vaccinated and non-vaccinated participants.

The link between conspiracy theory belief and vaccine hesitancy was consistently reported in literature on COVID-19 (Bertin et al., 2020; Bronstein et al., 2022; Romer and Jamieson, 2020) as well as the hypothesized path from paranoia to belief in conspiracy theories. Again, mistrust in science and healthcare professionals had already been reported as positively related with paranoia (Qunaibi et al., 2021) and negatively related to attitudes toward vaccines (Antinyan et al., 2021; Nazlı et al., 2022). These paths were also consistent with the previous model by Simione et al. (2021), who reported those two factors as the main predictors of vaccine intention. Consistent with Simione et al. (2021), paranoia was not a significant predictor of mistrust in medical information. This suggests that the mode of information delivery may be less influential than trait-like skeptical attitudes, highlighting the importance of considering stable psychological characteristics in public health interventions. Vaccine hesitancy would be more likely related to a trait-like attitude based on skepticism, paranoia, and mistrust, and further connected with fear of diseases (Bendau et al., 2021) and vaccines’ side effects (Amanna and Slifka, 2005) also at a deep, unconscious level (Simione et al., 2022). Consistently, the misinformation would be effective mainly for people already biased toward skeptical view about vaccination or medical science. A recent study supports this hypothesis, showing how people’s personal experiences with COVID-19 could change their attitudes toward vaccination but not the fear of vaccination (Duradoni et al., 2022).

The resistance to change, and the difficulty of penetrating into the anti-vax movements, also suggest another important effect: they seem to increase the hostility among people who are pro-vaccination (Rozbroj et al., 2019). People favorable to vaccination tends to perceive anti-vax individuals negatively, considering them as misinformed, selfish, and conspirational. For pro-vaccine people it is particularly difficult to empathize with anti-vax people and their feelings, even when vaccine hesitancy has been associated with concerns about vaccine safety due to the rapid development of the vaccine (Wang et al., 2021). We could speculate that such difficulties in reciprocal understanding between pro- and anti-vax people could further increase the sense of mistrust and paranoia that characterizes the attitude contrary to vaccination. Then, if communication from authorities and medical institutions seems to be limited, or ineffective in changing anti-vax attitudes, increased social cohesion might make a difference. In this sense, communication directed toward the pro-vaccination people could paradoxically be more effective in reducing vaccine hesitancy overall in the population. This hypothesis could be further investigated in future studies on this topic.

In partial agreement with the hypotheses, we did not observe significant effects of depression and anxiety on vaccine hesitancy and behavior, which is in contrast with previous studies (Bendau et al., 2021; Roberts et al., 2022; Simione et al., 2021). Regarding the death anxiety effect, a similar evaluation applies, as it is not consistent with the models proposed in Simione et al. (2021) and Scrima et al. (2022). However, the present findings are consistent with the result by Moscardino et al. (2022) who collected data in the same time window (spring 2021), while the other mentioned studies were based on data collected at the beginning of the pandemic (spring 2020) (Scrima et al., 2022; Simione et al., 2021) or later (Bendau et al., 2021; Roberts et al., 2022), but when the vaccines were not available to the general population. This highlights the dynamic interaction between situational factors and psychological predictors of vaccine behavior.

This study also has some limitations. First, convenience sampling introduced potential biases, including self-selection, sex imbalance and higher vaccination rates than the general population. Independent meta-analyses investigating the antecedents of COVID-19 vaccination have reported a gender effect on vaccine hesitancy (Baghani et al., 2023; Nehal et al., 2021), such as fewer women expressing the intention to receive the COVID-19 vaccine compared to men (Zintel et al., 2023). Additionally, considering the conflicting results on the effect of gender on conspiracy beliefs and mistrust (see van Mulukom et al., 2022), future studies should be conducted to further elucidate this pattern of relationship, which have not been properly explored even before the beginning of the COVID-19 pandemic (Dubé et al., 2018). Second, online administration and fixed item sequences could have affected responses. For example, the answers to BOC-19 items about conspiracy beliefs and mistrust in science could have influenced the latter evaluation of the COVID-19 vaccines. Third, the cross-sectional design precludes causal inference.

Building on the current findings, future studies should employ longitudinal and experimental designs to clarify the causal pathways between paranoia, conspiracy beliefs, mistrust, and vaccination behavior, including how these psychological traits influence the formation and persistence of vaccine attitudes over time, and the mechanisms through which social, informational, and environmental factors interact with individual predispositions to shape both intentions and actual vaccination uptake. Additionally, research could explore the dynamics between pro- and anti-vaccine groups, examining how mutual perceptions, social cohesion, and empathy affect vaccine attitudes and behavior. Investigating the moderating role of social networks, communication strategies, and pro-vaccine group influence could provide insights into effective interventions to reduce hesitancy. Lastly, experimental manipulations, such as inducing mortality salience (Farias et al., 2013) or using implicit association measures (Simione et al., 2022), may help disentangle the relative contribution of stable psychological traits, like paranoia and generalized mistrust, versus situational or contextual factors, such as exposure to misinformation, perceived threat, or social influence, in shaping vaccine attitudes and behavior. Expanding research in these directions will enhance theoretical understanding of distrust-related psychological mechanisms and offer practical guidance for public health strategies aimed at increasing vaccine uptake.

## 5 Conclusion

In this study, the results highlight how vaccine hesitancy is shaped by relatively stable psychological traits rather than transient emotional states or information exposure. Paranoid ideation consistently predicts mistrust and conspiracy beliefs, contributing to negative vaccine attitudes and refusal (van Prooijen and Douglas, 2018) in the context of COVID-19 infection in Italy. Addressing these stable traits may be crucial for designing effective public health strategies and future vaccination campaigns. This finding further suggests that vaccine hesitancy may be less responsive to short-term informational campaigns and more effectively addressed through long-term strategies that build institutional trust and social cohesion.

From a practical perspective, these results imply that interventions should focus on strengthening trust in scientific and medical institutions through transparent communication, community-based engagement, and credible role models. Campaigns emphasizing empathy and shared social identity, rather than confrontation, may help reduce polarization between pro- and anti-vaccine groups. Furthermore, public health efforts could benefit from targeting social networks and online communities where mistrust and conspiracy narratives circulate, addressing misinformation indirectly by promoting critical thinking and psychological resilience.

Despite some limitations, our study identifies paranoia as a stable psychological factor consistently associated with vaccine hesitancy. Implications include the need for interventions targeting broader social influence networks, not just direct communication with anti-vaccine individuals, and the potential benefit of fostering mutual understanding between pro- and anti-vaccine groups to reduce mistrust. Based on these results and on the reciprocal difficulties in understanding the pro-vax and anti-vax positions by the respective groups (Rozbroj et al., 2019), we speculate about the need for studying the reciprocal perceptions of such groups and about interventions to construct a common view about vaccination. By highlighting the persistence of distrust-related psychological traits even after vaccines became available, this study adds novel evidence on the stability of vaccine hesitancy mechanisms across pandemic phases. The challenge of overcoming vaccine hesitancy in future vaccination campaigns, and research on anti-vax attitudes, should start from this point, and move on.

## Data Availability

The raw data supporting the conclusions of this article will be made available by the authors, without undue reservation.
