# Supplementary material for: Paranoia affected COVID-19 vaccine refusal by increasing beliefs in conspiracy theories and mistrust of science
Source: Front Psychol. 2025 Dec 16;16:1718763. doi: 10.3389/fpsyg.2025.1718763 (PMC12750619; doi:10.3389/fpsyg.2025.1718763)
Supplement: Supplementary file 1 [file Table_1.DOCX]

Table S1. SEM estimated coefficients for models in control analyses.

| Model including Stress scale | | | | | | | | |
| --- | --- | --- | --- | --- | --- | --- | --- | --- |
| Path | | | b | SE | CI_lower_ | CI_upper_ | β | p |
| Depression | → | BCT | 0.02 | 0.07 | -0.12 | 0.15 | 0.02 | 0.82 |
| Anxiety | → | BCT | 0.01 | 0.09 | -0.16 | 0.18 | 0.01 | 0.92 |
| Death anxiety | → | BCT | -0.02 | 0.06 | -0.14 | 0.09 | -0.02 | 0.71 |
| Paranoia | → | BCT | 0.11 | 0.04 | 0.04 | 0.19 | 0.21 | <0.01 |
| Stress | → | BCT | -0.07 | 0.07 | -0.20 | 0.07 | -0.08 | 0.33 |
| Depression | → | MMS | 0.02 | 0.08 | -0.13 | 0.17 | 0.02 | 0.78 |
| Anxiety | → | MMS | -0.03 | 0.09 | -0.21 | 0.15 | -0.03 | 0.74 |
| Death anxiety | → | MMS | -0.03 | 0.06 | -0.14 | 0.08 | -0.03 | 0.62 |
| Paranoia | → | MMS | 0.10 | 0.04 | 0.02 | 0.17 | 0.19 | <0.05 |
| Stress | → | MMS | 0.04 | 0.07 | -0.10 | 0.18 | 0.05 | 0.54 |
| BCT | → | VacBehav | -0.11 | 0.02 | -0.15 | -0.08 | -0.34 | <0.01 |
| MMS | → | VacBehav | -0.12 | 0.02 | -0.16 | -0.08 | -0.34 | <0.01 |
| Depression | → | VacBehav | 0.02 | 0.02 | -0.02 | 0.06 | 0.06 | 0.39 |
| Anxiety | → | VacBehav | -0.01 | 0.02 | -0.06 | 0.03 | -0.03 | 0.57 |
| Death anxiety | → | VacBehav | 0.01 | 0.02 | -0.02 | 0.04 | 0.02 | 0.69 |
| Paranoia | → | VacBehav | 0.01 | 0.01 | -0.02 | 0.02 | -0.01 | 0.90 |
| Stress | → | VacBehav | 0.01 | 0.02 | -0.04 | 0.03 | -0.02 | 0.81 |
|  |  |  |  |  |  |  |  |  |
| Model including MMI scale | | | | | | | | |
| Path | | | b | SE | CI_lower_ | CI_upper_ | β | p |
| Depression | → | BCT | -0.02 | 0.06 | -0.14 | 0.10 | -0.02 | 0.73 |
| Anxiety | → | BCT | -0.02 | 0.08 | -0.18 | 0.14 | -0.02 | 0.82 |
| Death anxiety | → | BCT | -0.03 | 0.06 | -0.14 | 0.09 | -0.03 | 0.64 |
| Paranoia | → | BCT | 0.11 | 0.04 | 0.03 | 0.18 | 0.20 | <0.01 |
| Depression | → | MMI | 0.04 | 0.03 | -0.02 | 0.11 | 0.08 | 0.22 |
| Anxiety | → | MMI | -0.06 | 0.05 | -0.15 | 0.03 | -0.09 | 0.17 |
| Death anxiety | → | MMI | 0.04 | 0.03 | -0.02 | 0.10 | 0.07 | 0.25 |
| Paranoia | → | MMI | 0.03 | 0.02 | -0.01 | 0.08 | 0.11 | 0.13 |
| Depression | → | MMS | 0.04 | 0.07 | -0.09 | 0.18 | 0.05 | 0.52 |
| Anxiety | → | MMS | -0.01 | 0.09 | -0.19 | 0.16 | -0.01 | 0.87 |
| Death anxiety | → | MMS | -0.03 | 0.06 | -0.14 | 0.09 | -0.03 | 0.66 |
| Paranoia | → | MMS | 0.10 | 0.04 | 0.03 | 0.18 | 0.19 | <0.05 |
| BCT | → | VacBehav | -0.10 | 0.02 | -0.14 | -0.07 | -0.31 | <0.01 |
| MMS | → | VacBehav | -0.09 | 0.02 | -0.13 | -0.05 | -0.27 | <0.01 |
| MMI | → | VacBehav | -0.10 | 0.03 | -0.16 | -0.04 | -0.17 | <0.01 |
| Depression | → | VacBehav | 0.02 | 0.02 | -0.02 | 0.05 | 0.06 | 0.28 |
| Anxiety | → | VacBehav | -0.02 | 0.02 | -0.06 | 0.02 | -0.05 | 0.34 |
| Death anxiety | → | VacBehav | 0.01 | 0.02 | -0.02 | 0.04 | 0.03 | 0.51 |
| Paranoia | → | VacBehav | 0.01 | 0.01 | -0.02 | 0.02 | -0.01 | 0.84 |
|  |  |  |  |  |  |  |  |  |

Note. VacBehav = Vaccine behavior. b = unstandardized coefficient, SE = standard error, CI_lower_ and CI_upper_ = bootstrapped confidence intervals, β = standardized coefficient, p = significant level. Significant paths are reported in boldface. BCT = Belief in conspiracy theories, MMI = Mistrust in medical information, MMS = Mistrust of medicine and science.
